# Contractile Skeletal Muscle Cells Cultured with a Conducting Soft Wire for Effective, Selective Stimulation

**DOI:** 10.1038/s41598-018-20729-y

**Published:** 2018-02-02

**Authors:** Kuniaki Nagamine, Hirotaka Sato, Hiroyuki Kai, Hirokazu Kaji, Makoto Kanzaki, Matsuhiko Nishizawa

**Affiliations:** 1 Department of Finemechanics, Graduate School of Engineering, Tohoku University, 6-6-01 Aramaki, Aoba-ku, Sendai, 980-8579 Japan; 2 Department of Biomedical Engineering, Graduate School of Biomedical Engineering, Tohoku University, 6-6-04 Aoba-ku, Sendai, 980-8579 Japan

## Abstract

Contractile skeletal muscle cells were cultured so as to wrap around an electrode wire to enable their selective stimulation even when they were co-cultured with other electrically-excitable cells. Since the electrode wire was composed of the conducting polymer poly(3,4-ethylenedioxythiophene) (PEDOT) and polyurethane (PU), which is soft and highly capacitive (~10 mF cm^−2^), non-faradaic electrical stimulation with charge/discharge currents could be applied to the surrounding cells without causing significant damage even for longer periods (more than a week). The advantage of this new culture system was demonstrated in the study of chemotactic interaction of monocytes and skeletal muscle cells via myokines.

## Introduction

*In vitro* assays with an electrical stimulation system are required for quantitative investigations of the excitable cells such as neurons, cardiac cells, and skeletal muscle cells, as well as stem cell-derived organs and tissues in the near future^1–5^. Recent developments of organ-on-a-chip devices^6–8^ have inspired researchers to recapitulate the interactions of electrically-excitable cells with other organs in a closed perfusable microchannel. For example, recent studies showed that skeletal muscle functions as an endocrine organ that secretes a number of cytokines (myokines) in response to exercise in order to regulate whole body glucose metabolism, and that when this system is defective it leads to type 2 diabetes and obesity^9^. *In vitro* models such as organ-on-a-chip devices integrated with an electrical stimulation system will be beneficial for quantification of the exercise-triggered endocrine effects of myokines from skeletal muscles on the whole-body network of organs and tissues via the circulation.

Conventional electrical stimulation has been conducted using a pair of electrodes immersed away from the cells at either side of a culture chamber^4,5^ because the faradic reaction at stimulating electrodes often causes evolution of cytotoxic gas and pH change in the medium^10,11^. However, with such an electrode configuration (away from the cells), most of the ionic current flows past the medium without affecting the cells. More importantly, it is impossible, in principle, to selectively stimulate the desired group of muscle cells when they are co-cultured with other types of electrically-excitable cells such as neurons or monocytes. Direct combination of target cells and a stimulating electrode would solve the problem of selective stimulation, while this electrode should have a large electric double layer capacitance to avoid the cytotoxic faradaic reaction. Both carbon nanotube-based electrodes^12,13^ and conducting polymer-based electrodes^14–17^ have a large capacitance due to their large specific surface areas, and have been reported to be useful for low-invasive and site-selective stimulation of cells. However, previous studies used electrodes prepared on a solid planar substrate, on which it was difficult for contracting muscle cells to adhere onto the substrate over a long period of time^18^. Development of a site-selective electrical stimulation system compatible with contractile skeletal muscle cells will create an opportunity to investigate *in-vivo*-like electrophysiological activity of muscle cells as well as interaction of muscle cells with surrounding cells.

**Figure 1:**
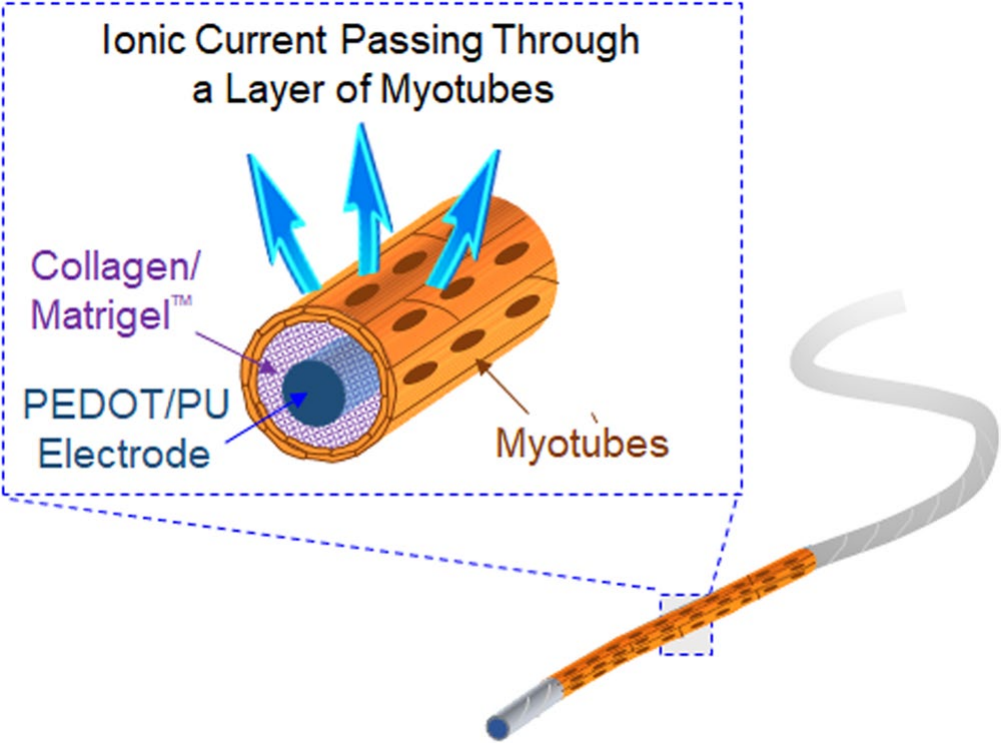
Schematic view of the skeletal muscle bundle, which is composed of a soft and highly-capacitive PEDOT/PU electrode wire wrapped inside a layer of contractile myotubes.

In the previous study^19,20^, we utilized a polymer composite consisting of conducting polymers poly(3,4-ethylenedioxythiophene) (PEDOT) and polyurethane (PU) to fabricate a soft organic electrode (PEDOT/PU composite electrode) that conformed to active motion of the tissue. In addition, the PEDOT/PU composite electrode retains large surface capacitance (~10 mF cm^−2^)^20^. In this study, this soft and highly capacitive electrode was processed into twisted fine wire, and then enveloped in a layer of contractile myotubes to form a skeletal muscle bundle (Fig. 1). The advantage of this new culture system was demonstrated by selective stimulations of the skeletal muscle bundle to study contraction-dependent endocrine effects of myokines on the activity of co-cultured monocytes.

## Results and Discussion

**Figure 2:**
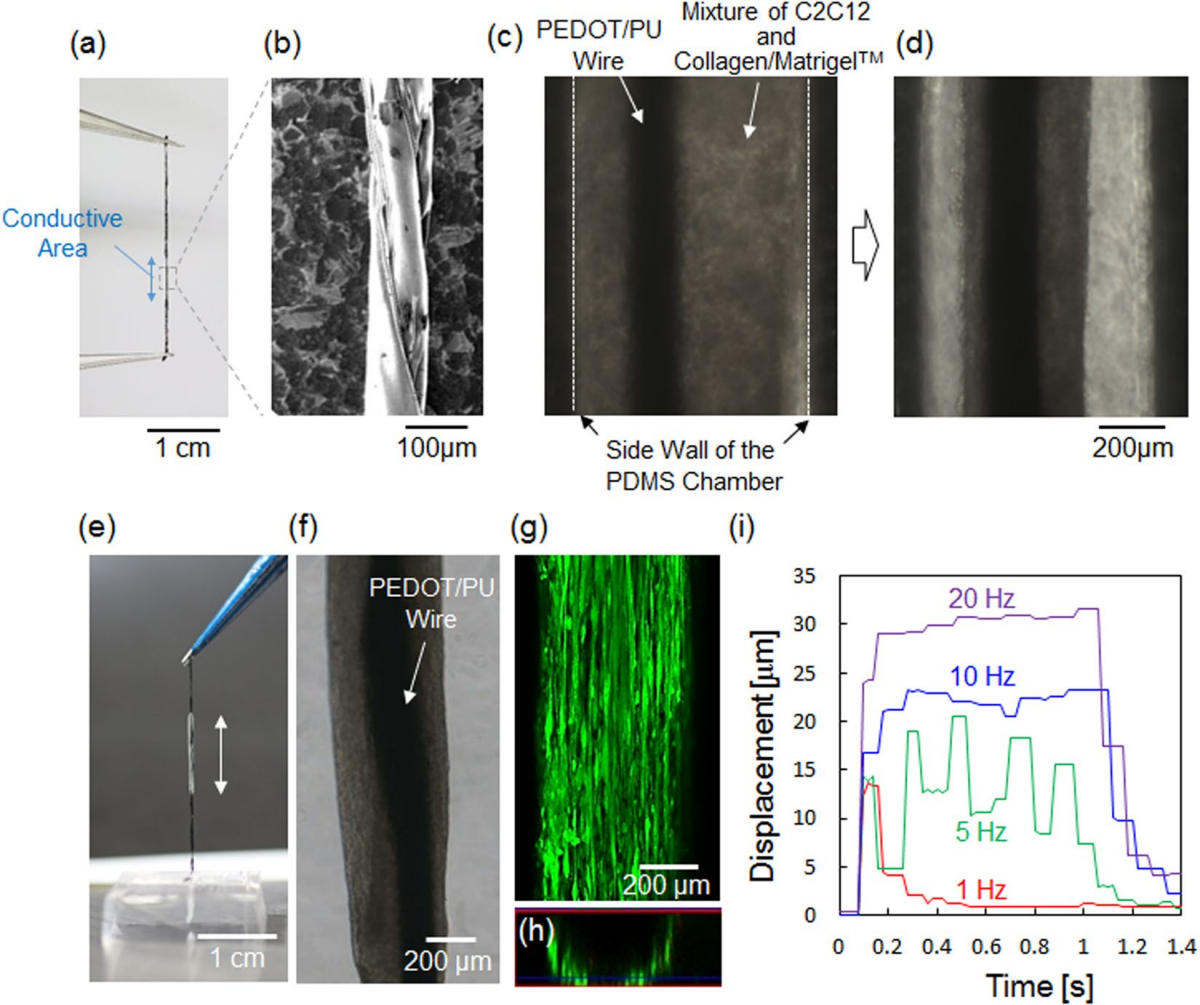
(**a**) Photograph of the PEDOT/PU wire held by tweezers. The double-headed blue arrow indicates the conductive area. (**b**) SEM image of the magnified view of the conductive area of the PEDOT/PU wire. (**c,d**) Phase contrast photomicrographs of the PEDOT/PU wire placed in the PDMS chamber with the myoblasts/hydrogel composite before (**c**) and after (**d**) 10 days of culture. (**e**) Photograph of the myotube-cultured PEDOT/PU wire lifted out from the PDMS chamber. The double-headed white arrow indicates the myotubes. (**f,g**) Phase-contract (**f**) and the corresponding fluorescent images (**g**) of the myotubes cultured around the PEDOT/PU wire. (**h**) The lower half of the cross-section of a Z-stack fluorescent image of (**g**). (**i**) Contractile displacement of the myotubes stimulated with an internal PEDOT/PU electrode wire against different frequency of the applied constant current pulses (5 mA amplitude, 10 ms duration, 50 μC charge injection).

Figure 2a and b show photographs of the twisted PEDOT/PU wire (diameter: ca. 190 μm) held by tweezers and its SEM image. The PEDOT/PU wire was insulated with virgin PU film, except the conductive area of 5 mm in length, indicated by the blue double-headed arrow (Fig. 2a). As can be seen in the SEM image in Fig. 2b, the PEDOT/PU wire had a grooved surface, and its twisted structure was stable in the medium probably because of the hydrogen bonding with the functional groups of N-H and C=O of urethane^21^. The charge storage capacity of the twisted PEDOT/PU wire was 59.8 ± 2.4 μC, calculated from the cyclic voltammogram by integrating the current over 0 V to 0.5 V (Supplementary Figure S1). This result ensures harmless electrical stimulation of the cells by the charge/discharge current within this capacity. The electrical resistance of the twisted PEDOT/PU wire (total length, 30 mm) was 2.55 ± 0.01 kΩ cm^−1^ (n = 3). Despite its relatively higher resistivity than metals, the temperature change of the electrode due to Joule heating was less than ± 0.5 °C (Supplementary Figure S2) during 20 h of continuous bipolar electrical stimulation, assuming harsh tetanic contractions (50 μC of constant current pulses, frequency: 200 Hz, train: 1 s, interval: 10 s) and twitch contractions (50 μC of constant current pulses, frequency: 1 Hz).

Figure 2c shows the PEDOT/PU wire set in the PDMS chamber (1 mm × 7 mm × 4 mm) with C2C12 myoblasts suspended in hydrogels of mixed collagen and Matrigel™. During the growth (three days) and the differentiation (seven days) periods of the myoblasts, the hydrogel contracted due to cell remodeling as shown in Fig. 2d^22^. The grooved structure of the PEDOT/PU wire resulted from twisting was found to be necessary to maintain the myotube/hydrogel composite. In fact, when we used a PEDOT/PU planar strip without twisting and a PEDOT-coated Au wire (200 μm in diameter), the myotube/hydrogel composite gradually detached and shrank along the long axis of the strip or wire (Supplementary Figure S3). These results suggested that the myotube/hydrogel composite shrank along the long axis of the PEDOT/PU wire with hanging to its grooved structure.

Figure 2e shows the skeletal muscle bundle lifted out from the PDMS chamber. The white area indicated by a double-headed white arrow is the myotubes. The microscopic image (Fig. 2f) shows that the bundle was composed of myotubes enclosing the electrode wire, with an overall outer diameter of approximately 500 μm. The fluorescent Live/Dead assay of the myotubes stained with calcein-AM and propidium iodide indicates cytocompatibility of the PEDOT/PU wire^19^ and alignment of myotubes along the long axis of the wire (Fig. 2g). A previous study reported that the myotubes can align along the extracellular matrix fibre aligned by uniaxial mechanical stretching^23^. Our findings showed that the myoblasts aligned along to the collagen/Matrigel™ matrix fiber shrinking along the long axis of the twisted PEDOT/PU wire. The confocal image of the cross-section (Fig. 2h) revealed the localization of the myotubes at the periphery of the hydrogel enclosing the PEDOT/PU wire, which was also confirmed with HE-staining of a slice of the myotubes enclosing the electrode wire (Supplementary Figure S4). These images suggested the myotubes were cultured not directly on the PEDOT/PU electrode wire, but on the surface of collagen/Matrigel™ matrix encapsulating the PEDOT/PU electrode wire. A similar structure was previously reported, where myotubes cultured in a cylindrical form of collagen gel^24^ where the myoblast cells migrated to the periphery of the collagen gel due to poor diffusion of nutrients and oxygen deeply into the matrix, and differentiated into the aligned and matured myotubes. Figure 2i shows the contractile behaviours of the myotubes upon application of constant current pulses of different frequencies (5 mA amplitude, 10 ms duration, 50 μC charge). The myotubes exhibited typical twitch contractions at low frequencies of 1 and 5 Hz, and tetanic contraction at high frequencies of 10 and 20 Hz as shown in Supplementary Movies S1 and S2, even when the cells were cultured around the soft electrode wire, suggesting maturation of sarcomere assembly in the myotubes. The cells continued twitch contractions at 1 Hz upon constant current pulse stimulations (5 mA amplitude, 10 ms duration, 50 μC charge) for more than 1 week without detachment from the electrode wire (Supplementary Figure S5), suggesting that faradaic reactions and joule heating resulted from the stimulation did not cause significant damage to the cells.

**Figure 3:**
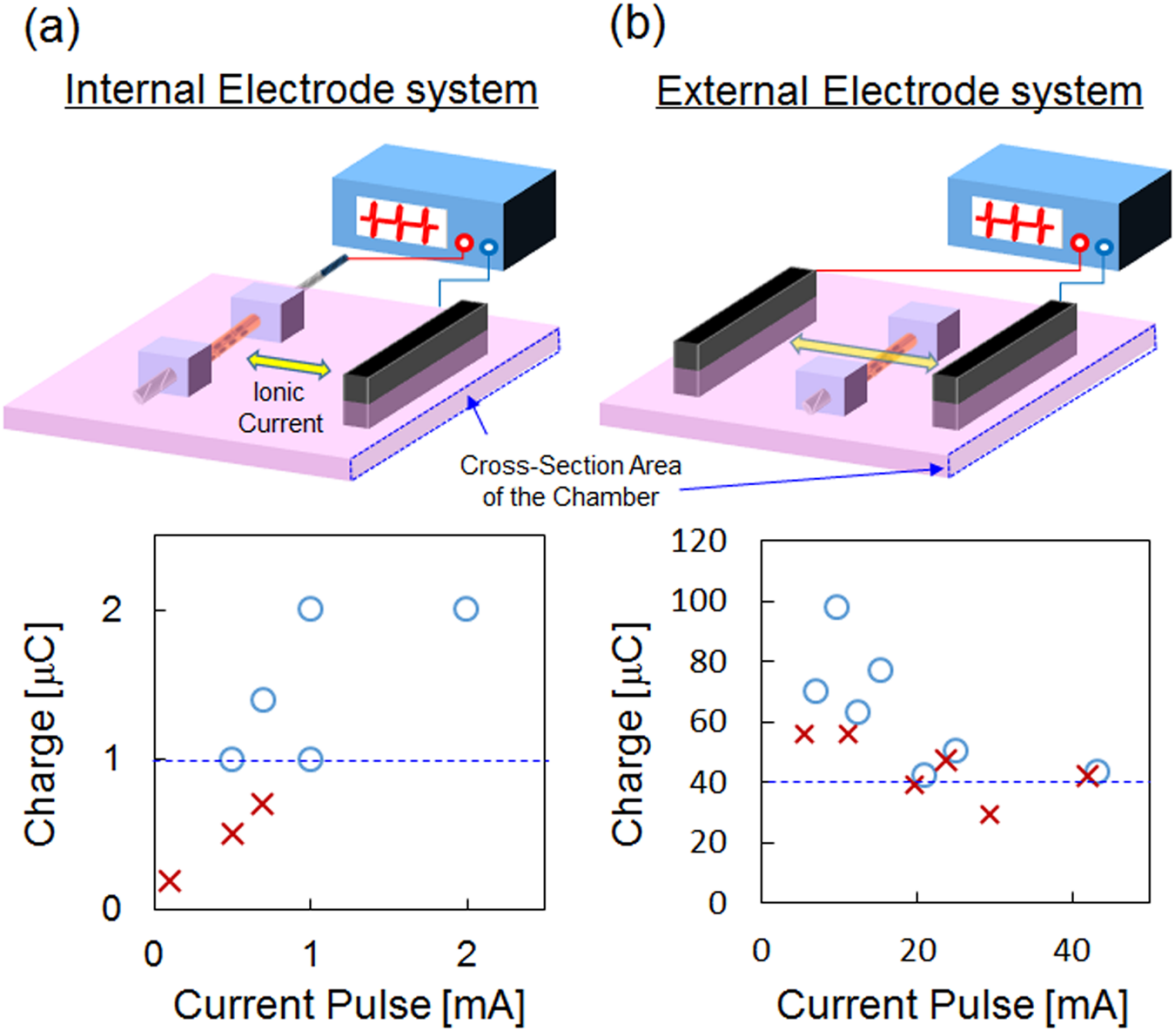
Plots of excitation (○) and non-excitation (×) of myotubes in the current-charge format. The myotubes cultured around the PEDOT/PU wire were stimulated using the internal (**a**) and external (**b**) electrode system as shown in the drawings. The blue dotted lines in the graphs show the judged threshold charge for excitation of the myotubes.

As shown in Fig. 3, the smallest charge required for myotube contraction was studied for stimulation with the enclosed PEDOT/PU electrode wire by means of previously developed technique^17^, and compared with the conventional system using a pair of external electrodes. The amplitude of the current pulse and pulse duration were changed, and the excitation was evaluated by the transient increase of intracellular Ca^2+^ in 80% of Fluo-4 AM-loaded myotubes in one observation field. In the case of Supplementary Movie S3, for example, the ratio of excited myotubes was calculated to be ca. 88% (total number: 33, excited cell number: 29). Figure 3a shows the current–charge plot of excitation (○) and non-excitation (×) of myotubes stimulated with the internal electrode wire. The current pulse with large charge injection tended to constitute an effective stimulation condition regardless of the current amplitude. The threshold charge could be roughly traced by the blue dotted line in the figure at 1 μC (Fig. 3a), whereas more than about 40 μC was required for conventional bulk stimulations using a pair of external electrodes (Fig. 3b). In this system, all the ionic current effectively flowed from the internal electrode wire to the external electrode through the layer of myotubes. The threshold charge density can then be calculated from the applied charge divided by the surface area occupied by the myotubes. The threshold charge density of the system of Fig. 3a was calculated to be 12.7 μC cm^−2^ from the 1 μC of charge divided by the surface area of the layer of myotubes of 0.08 cm^2^ (0.05 cm in diameter, 0.5 cm in length). On the other hand, excitation by the extracellular current pulse in the setup shown in Fig. 3b was inefficient, and therefore the threshold charge density for excitation was about 45 μC cm^−2^ which was calculated from the threshold charge 40 μC and the cross-sectional area of the chamber (0.9 cm^2^). Therefore, selective electrical stimulation could be achieved by stimulating the target cells using the encapsulated PEDOT/PU wire with a much smaller charge than the traditional external electrode techniques.

**Figure 4:**
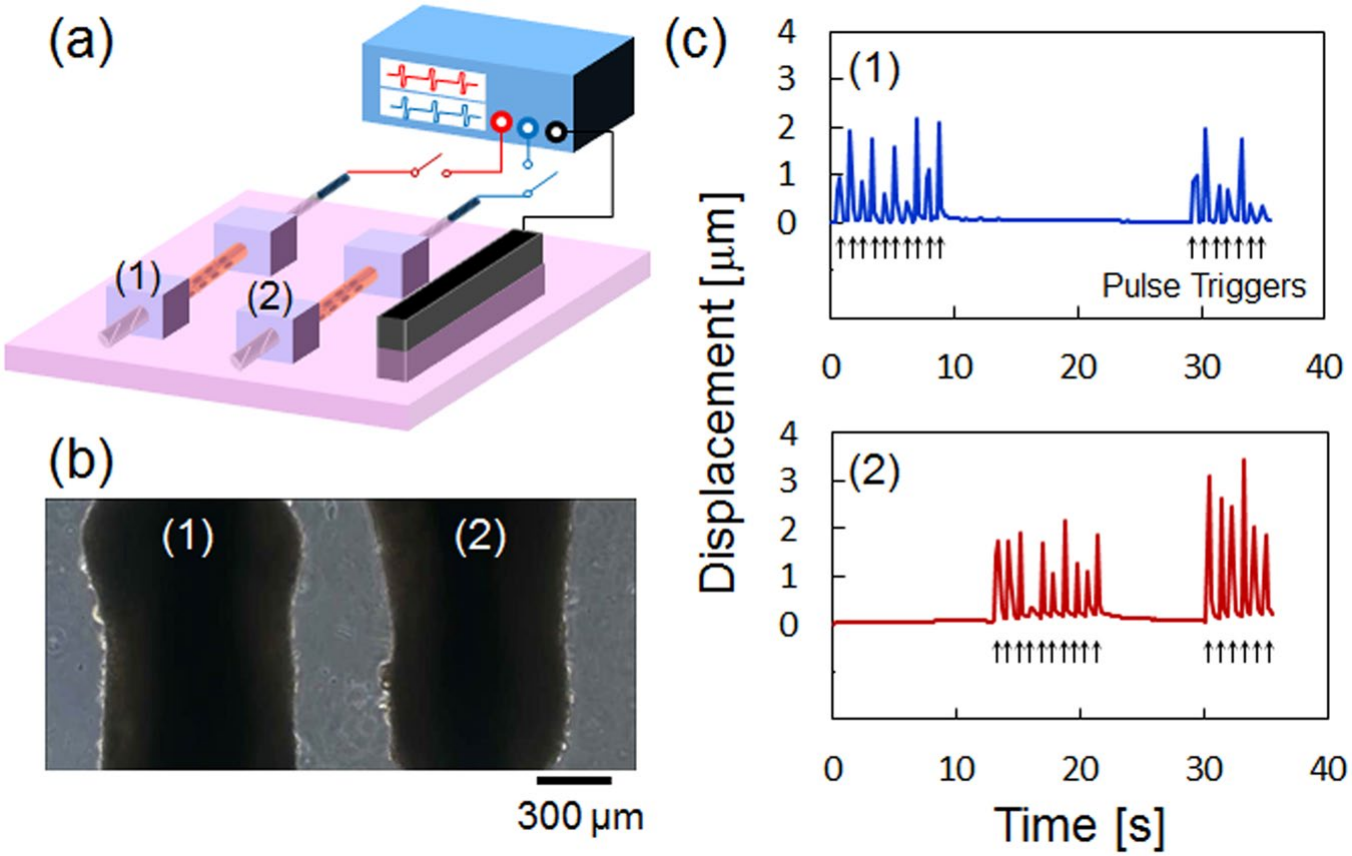
Selective stimulation of the arrayed skeletal muscle bundles enclosing PEDOT/PU electrode wires immersed in the same culture medium. (**a**) Schematic view of the experimental setup. (**b**) Phase-contrast microscopic view of the arrayed muscle bundles. (**c**) Time-course of contraction of muscle bundles 1 and 2 stimulated with periodic constant current pulses (0.23 mA amplitude, 10 ms duration, 2.3 μC charge injection, 1 Hz frequency). The arrows below the time course represent the trigger pulses.

Figure 4 shows the demonstration of selective stimulation of the arrayed skeletal muscle bundles. Portable skeletal muscle bundles could be handled with tweezers and placed 300 μm apart from each other as shown in Fig. 4a and b. One counter carbon electrode sharing the two internal electrode wires was set on one side of the culture dish (Fig. 4a). Figure 4c shows the time courses of contractile displacements of muscle bundles when stimulated with periodic bipolar constant current pulses (0.23 mA amplitude, 10 ms duration, 2.3 μC charge injection, 1 Hz frequency). Contractile displacement was determined from the real-time movie of muscle bundle contraction shown in Supplementary Movie S4. Pulse triggers were indicated by arrows below each time course. First, the left bundle (denoted as 1 in Fig. 4a) was selectively stimulated, followed by the right bundle (denoted as 2 in Fig. 4a). After a 10 s period of non-stimulation, the bundles were stimulated together. During stimulation of bundle 1 using this setup, the ionic current should flow through bundle 2 which was placed between internal electrode 1 and the external electrode. Even in this condition, bundle 2 did not show contraction because the charge density passing through it was 2.6 μC cm^−2^ (2.3 μC of charge divided by the cross-sectional area of the chamber), much lower than the threshold charge density for excitation by the external current pulse (45 μC cm^−2^) as identified in Fig. 3b.

**Figure 5:**
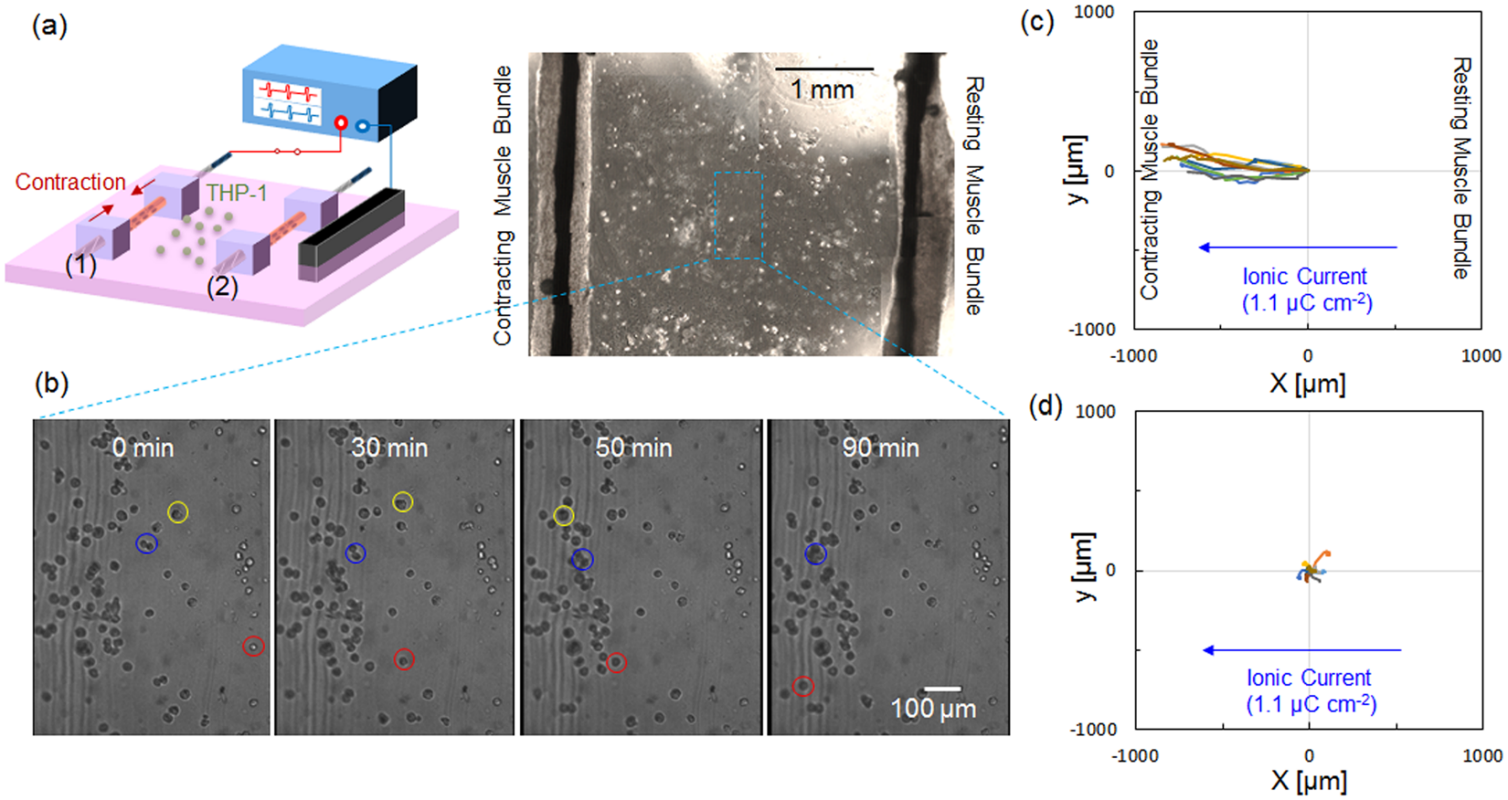
Demonstration of the monocyte chemoattraction assay using a THP-1/myotube co-culture system. (**a**) Schematic view of the experimental setup and the corresponding microscopic picture. (**b**) Snapshots taken from Supplementary Movie S5, which recorded the migration behaviour of the active three monocytes (marked as yellow, blue, and red circles) over 15 h of cultivation. Contracting myotubes were on the left side, while myotubes at rest were on the right side. (**c,d**) Spider plots of the migration of monocytes cultured with (**c**) or without (**d**) myotubes. In both cases, the bipolar constant current pulses (1 mA amplitude, 1 ms duration, 1 μC charge injection, 1 Hz frequency) were applied between the PEDOT/PU electrode wire-1 and the external carbon electrode.

Finally, this selective electrical stimulation system was applied to the co-culture system to evaluate monocyte chemoattraction induced by myokines secreted from contracting skeletal muscle cells. Miyatake *et al*. demonstrated that monocyte chemoattraction was induced by the addition of conditioned medium taken from the culture of contracting C2C12 myotubes^25^. Using the Boyden chamber assay, cells that vertically moved through the porous membrane along the concentration gradient of the chemoattractant were counted. This study suggests the possibility that skeletal muscles inflamed during active exercise secrete the chemoattractant into the circulation in order to generate macrophages through the infiltration of monocytes from the circulation. On the other hand, the Boyden chamber assay is limited in evaluating horizontal migration of monocytes toward chemoattractants secreted from contracting skeletal muscles. More importantly, monocytes, also known as electrotactic cells^26^, are activated by triggered calcium signalling^27^. We confirmed that the threshold charge required to induce a Ca^2+^ transient in THP-1 monocytes was around 4 µC (charge density passing through the culture medium: 4.4 μC cm^−2^) by using a pair of external carbon electrodes (Supplementary Figure S6). Therefore, to assess the dynamic migration behaviour of monocytes co-cultured with skeletal muscle cells, a system capable of stimulating the muscle cells alone is necessary. We consider that the co-culture assay proposed by Miyatake is appropriate to showing advantage of our present culture system. In our study, two skeletal muscle bundles were set 3 mm apart from each other. THP-1 monocytes were seeded between the two muscle bundles (Fig. 5a), and located in the position where the cells firstly adhered (at 0 min in Fig. 5b). Then, only the left side of the muscle bundle (denoted as 1 in Fig. 5a) was stimulated with bipolar constant current pulses (1 mA amplitude, 1 ms duration, 1 μC charge injection, 1 Hz frequency). Even under these conditions, muscle bundle 2 and THP-1 were not excited because the charge density passing through the culture medium, approximately 1.1 μC cm^−2^, was less than the excitation thresholds of both types of cells. Some THP-1 cells showed chemotactic migration, but most showed no response probably because of low viability or strong adhesion of the cells on the Matrigel™-coated dish. Figure 5b shows snapshots taken from Supplementary Movie S5, which recorded the migration behaviour of three active monocytes (marked as yellow, blue, and red circles) on the Matrigel™-coated dish during 15 h of cultivation. These monocytes migrated toward contracting muscle bundle immediately after application of bipolar constant current pulses, while there were no cells migrated toward the non-stimulated muscle bundle. Miyatake *et al*. measured the gradual increase of chemoattractants from contracting myotubes after around 2 h of continuous electrical stimulation^25^. Because our myotubes were pre-exercised overnight before the co-culture experiment, the myotubes were able to secrete chemoattractants immediately after applying electrical stimulations. Figure 5c shows the spider plots of a 1000 µm × 1000 µm area in which migrations of the most active ten monocytes in Supplementary Movie S5 were tracked using MtrackJ of ImageJ software. The (0, 0) position in the spider plot represents the positions where the cells adhered at first. The spider plot shows the directional migration of monocytes toward the contracting skeletal muscle bundle. On the other hand, no directional migration was observed in mono-culture of the ten THP-1 monocytes randomly selected in Supplementary Movie S6 using the same setup and pulse application (Fig. 5d), indicating that migration of the cells was due to chemotaxis, not electrotaxis. The average migration speed of the monocytes toward the contracting muscle bundle was calculated to be 5.1 ± 1.5 µm min^−1^ (n = 10) from the total migration distance divided by migration time. This migration speed was similar to that of THP-1 monocytes migrating toward a linear gradient of chemoattractant evaluated using a commercially-available chemotaxis assay system^28^. To our best knowledge, this is the first study in quantifying the migration speed of THP-1 monocytes toward co-cultured contracting skeletal muscle cells in real-time, which impossible in the Boyden chamber assay.

## Conclusions

In the present study, contractile myotubes were cultured so as to wrap around soft and highly capacitive PEDOT/PU wire for effective and selective electrical stimulation in a co-culture system. The twisted PEDOT/PU wire enabled stable anchorage of collagen/Matrigel™ on the periphery of which the myotubes were adhered, as well as low-invasive electrical stimulation with reversible charging/discharging ionic current for more than a week. This new culture system selectively controls the skeletal muscle contraction because the ionic current effectively passes through the desired type of cells even in a co-culture system, which is impossible in the conventional bulk electrical stimulation system using a pair of electrodes immersed in a medium. The advantage of the selective electrical stimulation system was successfully demonstrated by evaluating chemotaxis of monocytes toward co-cultured contracting skeletal muscle cells, although more samples should be evaluated for detailed statistical analysis in the future study. On the other hand, maturation of the myotubes was not fully verified in the present culture system. Physiological quantification of myotube maturation and further optimization of culture condition are necessary for advanced studies including the effect of skeletal muscle bundles structure on contractile activity, high-throughput evaluation of contractile dynamics in response to varied patterns or sub-threshold excitation, revealing mechanism of myokine-dependent THP-1 migration, quantitative *in vitro* assay of exercise-dependent interaction with other organs and tissues via myokine secretion, and myokine-dependent regulation of type2 diabetes and obesity using this organ-on-a-chip system. Furthermore, combination of the present system with a 3D printer and a microfluidic system will be promising techniques in providing complex physiological skeletal muscle tissue/electrode interface in future studies.

## Methods

### Cell culture

Cultivation of murine C2C12 myoblast cells (up to 7 passages; American Type Culture Collection, Manassas, VA, USA) has been reported previously^29,30^. Briefly, the myoblasts were cultured at 37 °C under a 5% CO_2_ atmosphere in growth medium composed of Dulbecco’s modified Eagle’s medium (DMEM, Wako Pure Chemicals Industries Ltd, Osaka, Japan) containing 10% fetal bovine serum (FBS, BioWest, Nuaillé, France), 100 U mL^−1^ penicillin, and 100 μg mL^−1^ streptomycin (Gibco, Thermo Fisher Scientific, Waltham, MA, USA). When cell densities reached approximately 70% confluence, the cells were detached with 0.25% trypsin/0.01% EDTA (Gibco, Thermo Fisher Scientific) and were either replated or used in experiments. THP-1 monocytes (American Type Culture Collection) were grown for three days in RPMI 1640 (Gibco, Thermo Fisher Scientific) containing 5% FBS and 100 U mL^−1^ penicillin, 100 μg mL^−1^ streptomycin^25^, and were either replated or used in experiments.

### Fabrication of the PEDOT/PU wire

**Figure 6:**
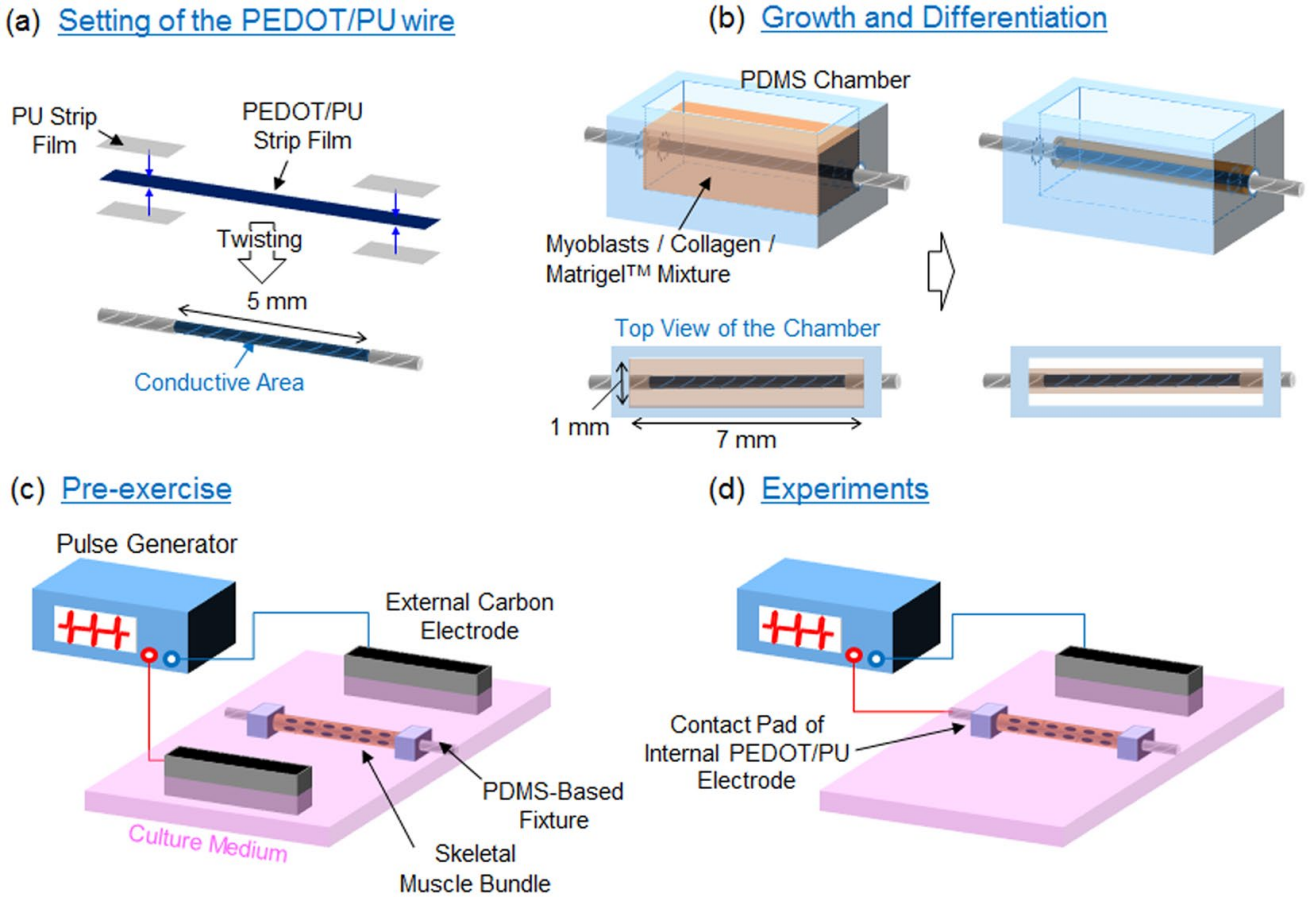
The process of skeletal muscle cell culture around the PEDOT/PU wire. (**a**) A PEDOT/PU film strip was sandwiched between the virgin PU strip films while maintaining exposure of a conductive area, followed by twisting to form a wire. (**b**) Set-up for cultivation of muscle cells. The PEDOT/PU wire was attached to the PDMS chamber with the myoblasts/collagen/Matrigel™ mixture. After gelation of the mixture, the cells were grown for 3 days and differentiated for an additional 7 days in the medium. (**c**) Setup for pre-exercise. The skeletal muscle bundle was set in the 4-well dish and electrically stimulated overnight with a pair of carbon electrodes to induce cellular contractile activity. (**d**) Experimental setup for electrical stimulation of the skeletal muscle bundle. Constant current pulses were applied between the internal PEDOT/PU electrode wire and the external carbon electrode to induce contraction of the myotubes.

The PEDOT/PU films (3 μm in thickness) were prepared as described previously^19,20,31^. Briefly, the precursor mixture solution composed of butanol (2 mL), EDOT (0.88 mL of 1 M solution), pTS Fe(III) (6.5 mL of 0.4 M solution) and PU/THF solution (10 wt%, 35 mL) was spin-coated for 30 s at 750 rpm, followed by thermal polymerization of PEDOT at 100 °C for 10 min. The film was washed overnight with distilled water to remove cytotoxic molecules. The PEDOT/PU film on a glass substrate was cut into a long strip (30 mm × 3 mm) using a laser processing machine (VLS3.50, Universal Laser Systems GMBH, Vienna, Austria). As shown in Fig. 6a, the PEDOT/PU strip was sandwiched between the virgin PU strip films (13 mm × 4 mm, 3 μm in thickness) to insulate the strip while exposuring a 5 mm length of conductive area. Finally, the assembled strip was twisted 10 times to create the PEDOT/PU wire.

### Preparation of skeletal muscle bundles

A PDMS chamber (1 mm in width, 7 mm in length, 4 mm in depth) with through holes (1 mm in diameter) at both side of the walls was sterilized by UV irradiation, and treated with 0.2% (w/v) pluronic F-127 solution (Thermo Fisher Scientific) for 1 h at room temperature to prevent adhesion of cells and gel to the inner wall of the PDMS chamber. Figure 6b shows the setup for cultivation of muscle cells, inside which the PEDOT/PU wire was placed together with a 10 μL of solution containing a cell suspension (10^7^ cells mL^−1^) of C2C12 myoblasts, neutralized collagen type I solution (Nitta Gelatin, Osaka, Japan), and Matrigel™ (Corning, Corning, NY, USA) in a volume ratio of 3:6:1. As can be seen in the top view of the chamber (Fig. 6b), the PEDOT/PU wire including its insulating area was inserted into the chamber so that the cells were able to fully cover the conductive area of the wire. The setup was incubated for 30 min at 37 °C under a 5% CO_2_ atmosphere to facilitate gelation of the mixture, followed by three days of culture in the growth medium until the cells were fully confluent. The myoblasts were then induced to differentiate into myotubes for an additional seven days by replacing the growth medium with differentiation medium composed of DMEM, 2.0% calf serum (Thermo Fisher Scientific), 1.0 nM insulin (Sigma Aldrich, St Louis, MO, USA), 100 U mL^−1^ penicillin, and 100 μg mL^−1^ streptomycin. The differentiated cells were then pre-exercised using the setup shown in Fig. 6c. The skeletal muscle bundle was transferred to the center of a 4-well multi cell culture dish (30 mm × 80 mm, Nunc, Thermo Fisher Scientific) with a pair of carbon electrodes at either side of the dish (IonOptix, Dublin, Ireland). Both ends of the skeletal muscle bundle were fixed to the PDMS-based cubic fixtures previously fixed in the dish. The skeletal muscle bundles were pre-exercised overnight by applying periodic pulses (0.7 V mm^−1^ amplitude, 1 Hz frequency, 2 ms duration) with a pair of carbon electrodes, which were previously optimized to induce sarcomere assembly and cellular contractile activity in the EPS medium composed of phenol red-free DMEM containing 2.0% calf serum, 100 U mL^−1^ penicillin, and 100 μg mL^−1^ streptomycin^32^.

### Constant current pulse stimulation of the contractile skeletal muscle bundles

Figure 6d shows the setup for electrical stimulation of the myotubes using the internal PEDOT/PU electrode wire. After pre-exercise, the contractile skeletal muscle bundle was carefully transferred to another 4-well culture dish and both ends of the bundle were fixed using PDMS-based fixtures. For the selective stimulation experiment, two skeletal muscle bundles were placed in the dish to be 0.3 mm apart from each other. One channel of the electrical pulse stimulator (SEN-7203, Nihon Kohden, Tokyo, Japan) coupled with an isolator unit (Nihon Kohden) was connected to the contact pad of the internal PEDOT/PU electrode wire extended from one end of the muscle bundle, and the other channel was connected to an external carbon electrode set to one side of the dish. Constant current pulses were applied between the internal PEDOT/PU electrode wire and the external carbon electrode to induce cellular contraction. Contractile displacement was tracked by using the motion analyser software (Keyence). Five reference points (indicated by black arrows in Supplementary Figure S7, for example) were selected in one image and their motion was tracked. The displacement was calculated by averaging motions of five reference points. For calcium imaging, the skeletal muscle bundle was immersed in serum- and phenol red-free DMEM containing the fluorescent calcium indicator, Fluo-4 (Dojindo, Kumamoto, Japan), for 30 min at 37 °C. Serum was removed because it significantly increases background fluorescence and decreases the S/N ratio. The cells were imaged with excitation at 488 nm and emission at 530 nm with a time lapse. The number of excited myotubes in the Fluo-4-loaded cells was counted from the image which was prepared by calculating the difference of fluorescent intensity of the cells before and after applying electrical stimulation.

### Selective electrical stimulation of skeletal muscle bundles co-cultured with monocytes

The surface of the 4-well dish was pre-treated with Matrigel™ diluted 10-fold in DMEM for 1 h at 37 °C to induce adhesion of THP-1 monocytes. Pre-exercised and non-exercised skeletal muscle bundles were placed in the center of the dish 3 mm apart from each other. THP-1 monocytes were then seeded between the two muscle bundles and incubated for 1 h at 37 °C to allow adhesion of the cells on the Matrigel™-coated surface. One of the muscle bundles was electrically stimulated by applying a bipolar constant current (current amplitude: 1 μA, frequency: 1 Hz, duration: 1 ms) in the EPS medium. The migratory behaviour of the monocytes was recorded using a real-time cultured cell monitoring system (CCM-1.4, ASTEC) in the time-lapse mode (10 min interval), and the results are shown as spider plots.

### Data availability statement

All data generated or analysed during this study are included in this published article and its Supplementary Information files.

## Electronic supplementary material

Supplementary Information

Movie S1

Movie S2

Movie S3

Movie S4

Movie S5

Movie S6
