# Supplementary material for: Contractile Skeletal Muscle Cells Cultured with a Conducting Soft Wire for Effective, Selective Stimulation
Source: Sci Rep. 2018 Feb 2;8:2253. doi: 10.1038/s41598-018-20729-y (PMC5797109; doi:10.1038/s41598-018-20729-y)
Supplement: Supplementary file 1 — Supplementary Information [file 41598_2018_20729_MOESM1_ESM.pdf]

Contractile Skeletal Muscle Cells Cultured with a Conducting Soft Wire for Effective, Selective Stimulation

Kuniaki Nagamine, Hirotaka Sato, Hiroyuki Kai, Hirokazu Kaji, Makoto Kanzaki, and Matsuhiko Nishizawa*

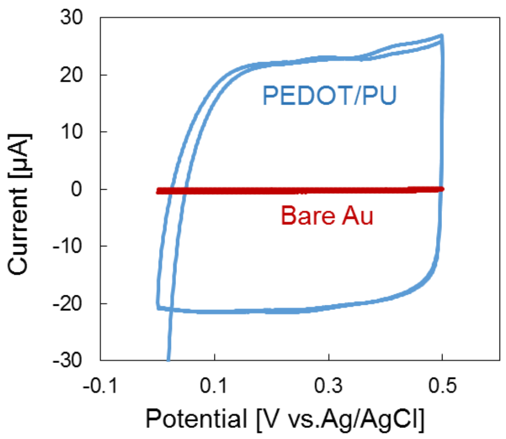

Figure S1. Cyclic voltammogram measured with the PEDOT/PU electrode wire in PBS solution. As compared to a bare Au electrode shown in the same figure, a larger capacitive current was obtained using the PEDOT/PU electrode.

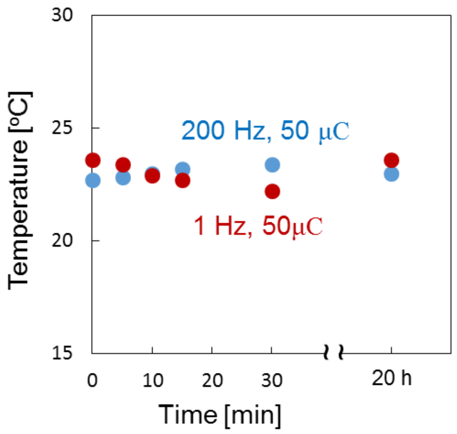

Figure S2. Surface temperature change of the PEDOT/PU wire immersed in medium at room temperature during application of periodic current pulses (Blue dots: 50 μC of constant current pulses, frequency: 200 Hz, train: 1 s, interval: 10 s. Red dots: 50 μC of constant current pulses, frequency: 1 Hz). Surface temperature was measured using an infrared thermometer (model SK-8900, skSATO).

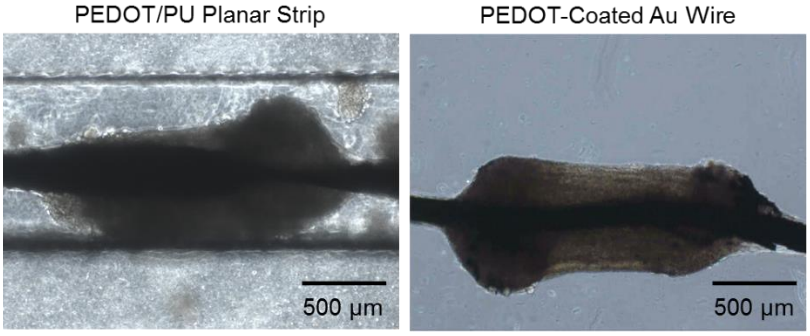

Figure S3. Phase-contrast photomicrographs of the myotube/hydrogel composite detached from a PEDOT/PU planar strip and a PEDOT-coated Au wire.

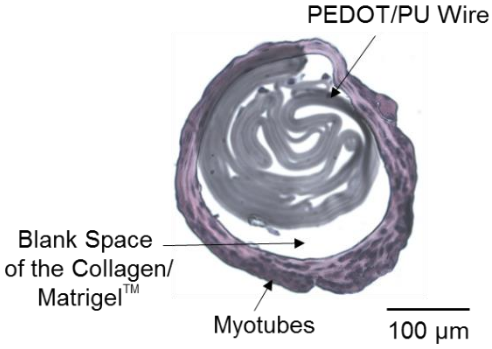

Figure S4. Cross-section of the myotubes enclosing the PEDOT/PU electrode wire stained with hematoxylin and eosin (H&E).

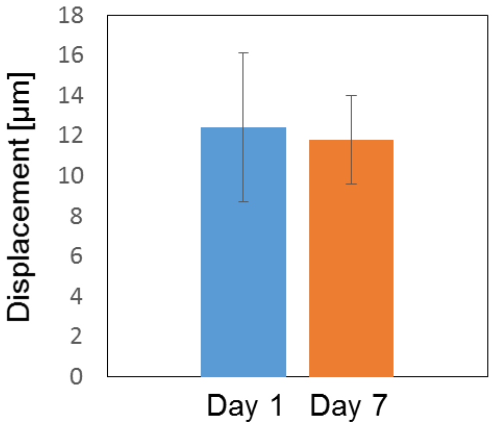

Figure S5. Contractile displacement of the myotubes after 1 and 7 days of stimulation with periodic constant current pulses (5 mA amplitude, 10 ms duration, 50 µC charge injection, 1 Hz frequency) with enclosed PEDOT/PU electrode wire (n = 3).

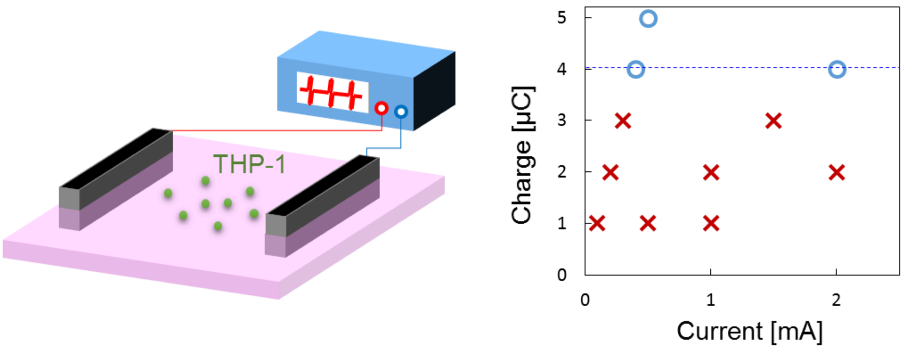

Figure S6. The plot of excitation (○) and non-excitation (×) of THP-1 monocytes in the current-charge format. For calcium imaging, THP-1 monocytes were previously immersed in serum- and phenol red-free DMEM containing the fluorescent calcium indicator, Fluo-4 for 30 min at 37°C. THP-1 monocytes spread in the 4-well dish were stimulated using a pair of carbon electrodes as shown in the drawing, and their fluorescent response was observed. The blue dotted lines in the graphs show the judged threshold charge for excitation of THP-1 monocytes.

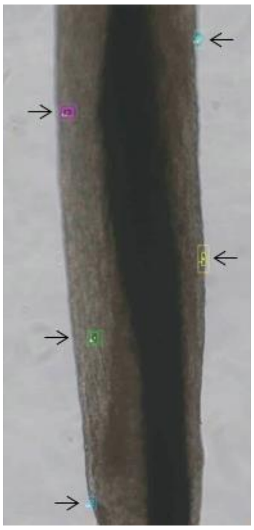

Figure S7. Phase-contrast micrograph of the skeletal muscle bundle. For measuring contractile displacement, five reference points (suggested as black arrows) were selected in one image and their motion was tracked using motion analyzer software (Keyence).

Supplementary Information

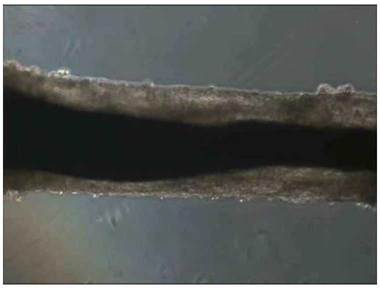
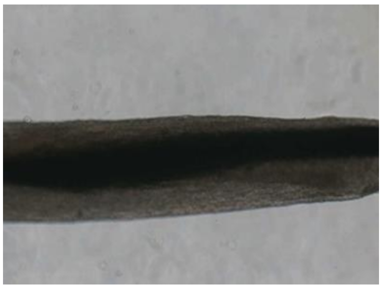

Movie S1 and S2 Contraction of the myotubes stimulated with internal PEDOT/PU electrode wire (5 mA amplitude, 10 ms duration, 50 μC charge injection) at 1 (S1) and 20 Hz (S2).

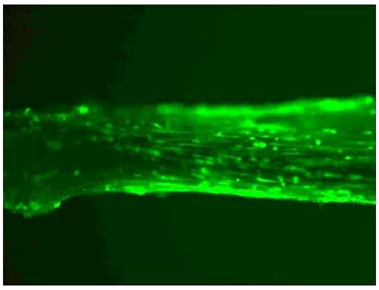

Movie S3 Contraction of the myotubes stimulated with internal PEDOT/PU electrode wire (1 mA amplitude, 1 ms duration, 1 μC charge injection, 1 Hz frequency). The cells were stained with the fluorescent calcium indicator, Fluo-4 AM.

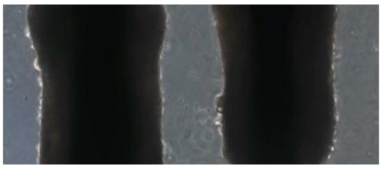

Movie S4 Selective stimulation of the arrayed skeletal muscle bundles.

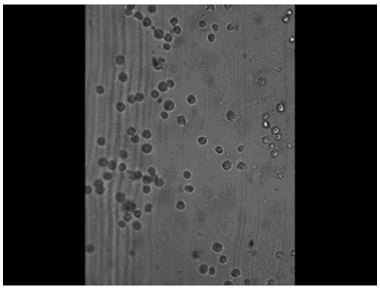
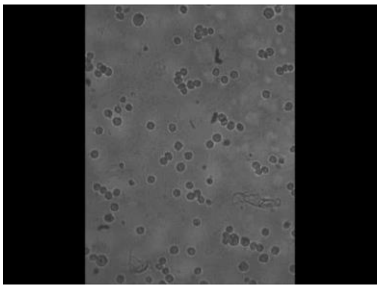

Movie S5 and S6 Migration of monocytes cultured with (S5) or without (S6) skeletal muscle bundles. In S5, the contracting muscle bundle was on the left side, while the resting muscle bundle was on the right side. In both cases, bipolar constant current pulses (1 mA amplitude, 1 ms duration, 1 μC charge injection, 1 Hz frequency) were applied between the internal PEDOT/PU electrode wire 1 and the external carbon electrode as shown in Fig. 5a.
